# Exact Inference for Hardy-Weinberg Proportions with Missing Genotypes: Single and Multiple Imputation

**DOI:** 10.1534/g3.115.022111

**Published:** 2015-09-15

**Authors:** Jan Graffelman, S. Nelson, S. M. Gogarten, B. S. Weir

**Affiliations:** * Department of Statistics and Operations Research, Universitat Politècnica de Catalunya, 08028 Barcelona, Spain; † Department of Biostatistics, University of Washington, Seattle, Washington 98105-9461

**Keywords:** Hardy−Weinberg equilibrium, missing data, imputation, exact test

## Abstract

This paper addresses the issue of exact-test based statistical inference for Hardy−Weinberg equilibrium in the presence of missing genotype data. Missing genotypes often are discarded when markers are tested for Hardy−Weinberg equilibrium, which can lead to bias in the statistical inference about equilibrium. Single and multiple imputation can improve inference on equilibrium. We develop tests for equilibrium in the presence of missingness by using both inbreeding coefficients (or, equivalently, χ^2^ statistics) and exact *p*-values. The analysis of a set of markers with a high missing rate from the GENEVA project on prematurity shows that exact inference on equilibrium can be altered considerably when missingness is taken into account. For markers with a high missing rate (>5%), we found that both single and multiple imputation tend to diminish evidence for Hardy−Weinberg disequilibrium. Depending on the imputation method used, 6−13% of the test results changed qualitatively at the 5% level.

Modern genotyping platforms produce large databases with information on tremendous numbers of genetic markers used in gene−disease association studies. Typically, such data sets contain a considerable number of missing observations; we will refer to such instances as “missings.” The missing data problem pervades much of the subsequent statistical analysis of the data. There are several approaches to deal with missing genotype data, as we briefly sketch here. First, the simplest and often-used approach is just to ignore the missings and exclude these from the computations. For very small amounts of missing data, this action may be justified, because omitting a few missings in a large data set is unlikely to affect the results of the study (Schafer 1997). However, if the number of missings is substantial, then there is a loss of power because of a reduced sample size if the missings are ignored. Moreover, if missings are not missing completely at random, then ignoring missings may lead to severe bias in the results of the analysis. In recent work (Graffelman *et al.* 2013) we have shown that the conclusions of basic tests on Hardy−Weinberg equilibrium (HWE) can be mistaken if the missings simply are ignored. On the other hand, we also have shown (Weir 2013) that the proportion of HWE rejections can be much closer to nominal in large-scale datasets if single-nucleotide polymorphisms (SNPs) with any missings are removed from consideration.

Second, missings can be imputed at the time the data are phased (Delaneau *et al.* 2014) or by the use of external reference panels (Howie *et al.* 2009). In these approaches the missing values often are inferred once, and a single completed data set is then used in the subsequent statistical analyses. The imputed data set is being treated as if it was a completely observed data set. Reference panels have become a popular tool for imputing missing genotypes (Howie *et al.* 2009). However, one cannot be sure that the imputed values are the correct ones. Taking the imputed data set as being the truly observed genotypes ignores the uncertainty about the imputations in posterior analyses.

Third, one can use multiple imputation (Little and Rubin 2002) of the missing data by using statistical models that borrow information from correlated variables to impute the missings. This approach takes the uncertainty in the imputations into account. The goal of this approach is not to impute the missing genotypes once and for all, but to do correct statistical inference on the genetic parameters of interest. The missing genotypes are imputed many times, leading to many completed data sets. Each completed data set is analyzed separately by the method of interest, and then the results are combined.

In this paper, we focus on multiple imputation as a tool for treating missings in genetic data analysis and use that approach to test markers for HWE. In genetic association studies tests for HWE are used as a tool for detecting genotype error (Gomes *et al.* 1999, Hosking *et al.* 2004, Laurie *et al.* 2010). In previous work (Graffelman *et al.* 2013) we described multiple imputation for inference on HWE based on the inbreeding coefficient. We argue below that the use of inbreeding coefficients amounts to using the χ^2^ test statistic for HWE. Today, the state-of-the-art test for HWE is the exact test (Wigginton *et al.* 2005; Rohlfs and Weir 2008) as this is the most powerful test. The purpose of this paper is to extend the previous results on inference with missings for HWE to exact test procedures for HWE.

Single imputations based on reference panels like the HapMap project (The International Hapmap Consortium 2007) or 1000 Genomes (The 1000 Genomes Project Consortium 2010) or produced by SHAPEIT (Delaneau *et al.* 2014) have become important tools in statistical genetics. We therefore dedicate some attention to comparing results obtained by multiple imputation with those obtained by using single imputations made by SHAPEIT.

The structure of the remainder of this paper is as follows. First we outline our multiple imputation approach for inference on HWE with missings. Then we describe a database from the GENEVA project (Boyd *et al.* 2009; Ryckman *et al.* 2012; Alleman *et al.* 2012) that we use as an example for our methodology and we present the results of a HWE analysis for a subset of SNPs from this project. The *Discussion* section completes this paper. References for software implementing the multiple imputation procedures outlined are provided.

## Materials and Methods

In this section we provide the basic notation and describe two approaches for inference on HWE when there are missing genotypes: single imputation (SI) and multiple imputation (MI). We restrict our attention to biallelic genetic markers with alleles A and B and let *p_A_* and *p_B_* be their respective allele frequencies. We define *n_AA_*, *n_AB_*, *n_BB_*, and *n_A_,n_B_* as the respective genotype and allele counts, and *n* as the total sample size.

### Single imputation

Estimation of missing genotypes in genetic studies is linked to the problem of haplotype estimation. If the haplotypes of an individual are known or have been estimated, then the missing values of SNPs that form part of that haplotype can in principle be inferred. There are several computer programs available for imputation of missing genotypes such as SHAPEIT2 (Delaneau *et al.* 2014), MaCH (Li *et al.* 2010), IMPUTE2 (Howie *et al.* 2012), or Beagle (Browning and Browning 2014). For imputation, haplotypes from HapMap (The International Hapmap Consortium 2007) populations or the 1000 Genomes Project (The 1000 Genomes Project Consortium 2010) are used as a reference panel. A comparison of these methods in terms of accuracy and required computational resources has been made (Howie *et al.* 2009) and is beyond the scope of the current paper. In this paper we adhere to SHAPEIT2 as a haplotype inference software for phasing complete chromosomes and use the results from this program to obtain single imputations of the missing genotype data.

### Multiple imputation

MI is a statistical tool for dealing with missing values (Little and Rubin 2002). It requires a statistic that can be calculated for each imputed dataset. If HWE is tested by the classical χ^2^ test, then multiple imputation can be performed with inbreeding coefficients (Graffelman *et al.* 2013). If the exact test is used for testing HWE, then multiple imputation can be performed using *p*-values from a one-sided exact test. We now sketch both these approaches.

#### Inbreeding coefficients:

Hardy−Weinberg disequilibrium can be parameterized by using the within-population inbreeding coefficient *f* (Crow and Kimura 1970; Weir 1996), and under this parameterization the population genotype frequencies are given by

$$\begin{array}{l} P_{AA}=p_{A}^{2}+p_{A}p_{B}f,\\ P_{AB}=2p_{A}p_{B}\left(1-f\right),\\ P_{BB}=p_{B}^{2}+p_{A}p_{B}f, \end{array}$$ (1)

with $-p_{m}/\left(1-p_{m}\right)\leq f\leq 1$, where *p_m_* is the minor allele frequency min(*p_A_*,*p_B_*). If *f* = 0, then the genotype frequencies correspond to the Hardy−Weinberg proportions. For *f* > 0 there is a deficiency of heterozygotes, and for *f* < 0, there is an excess. The inbreeding coefficient *f* can be estimated by maximum likelihood (ML) by the use of the multinomial distribution for genotype counts, and the ML estimator and its variance are given by the following (Weir 1996):

$$\hat{f}=\frac{4n_{AA}n_{BB}-n_{AB}^{2}}{n_{A}n_{B}}\;\text{and}\;\text{Var}\left(\hat{f}\right)=\frac{{\left(1-f\right)}^{2}\left(1-2f\right)}{n}+\frac{f\left(1-f\right)\left(2-f\right)}{2np_{A}\left(1-p_{A}\right)}.$$ (2)

We note that the ML estimator is related to the classical χ^2^ test statistic for HWE by $X^{2}=n{\hat{f}}^{2}$. For imputation we use a multinomial logit model that uses allele intensities and/or flanking markers as predictors. Imputed data sets are generated by the MICE algorithm (van Buuren and Groothuis-Oudshoorn 2011; van Buuren 2012). Multiple imputation yields a set of *m* complete data matrices of genotype information. To be able to perform statistical inference for HWE, inbreeding coefficients and their variances are estimated for all imputed data sets, and these estimates are combined according to Rubin’s pooling rules (Rubin 1987; Little and Rubin 2002). If ${\hat{f}}_{i}$ is the estimate of *f* from the *i*th of *m* imputations, we write

$$\bar{f}=\frac{1}{m}\sum_{i=1}^{m}{\hat{f}}_{i},\;\;W=\frac{1}{m}\sum_{i=1}^{m}\text{Var}\left({\hat{f}}_{i}\right),$$ (3)

where *W* is called the average within-imputation variance. Next, the between-imputation variance (*B*) and the total variance (*T*) are computed as

$$B=\frac{1}{m-1}\sum_{i=1}^{m}{\left(\hat{f_{i}}-\bar{f}\right)}^{2},\;\;T=W+\left(1+1/m\right)B.$$ (4)

A test statistic for HWE (*H*_0_: *f* = 0) is then given by $Q=\bar{f}/\sqrt{T}$. Under the null, this statistic has a *t_v_* distribution with ν degrees of freedom, ν given by

$$\nu =\left(m-1\right){\left(1+\frac{mW}{\left(m+1\right)B}\right)}^{2}.$$ (5)

The multiple imputation *p*-value, denoted by *p_mi_*, for a two-sided test for HWE after multiple imputation is given by

$$p_{\text{mi}}=2\text{Pr}\left(t_{\nu }\geq \left|Q\right|\right).$$

Alternatively, inference can be performed by calculating a 100(1 − *a*)% confidence interval given by $\bar{f}\pm t_{\nu ,1-\alpha /2}\sqrt{T}$. We note that $V\left(\hat{f}\right)$ in equations (2) and (3) may be calculated by substitution of $\hat{f}$ and ${\hat{p}}_{A}$, but we do not recommend this. Under the null hypothesis of *f* = 0, it follows that $\text{Var}\left(\hat{f}\right)=1/n$. If the sample estimates $\hat{f}$ and ${\hat{p}}_{A}$ are used, then $\text{Var}\left(\hat{f}\right)$ is often below 1/n, yielding a Wald statistic that is too liberal. We also point out that substitution of the sample estimate $\hat{f}$ yields a zero variance for samples that do not contain heterozygotes, because in that case we have $\hat{f}=1$. For markers with a low MAF, samples without heterozygotes can easily arise. Typically such samples have one homozygote with a low count and the other one with a high count. We note further that a zero homozygote count also puts the estimate $\hat{f}$ on the boundary of the parameter space because if *n_AA_* = 0, then $\hat{f}=-p_{A}/\left(1-p_{A}\right)$, and the latter coincides with the lower bound for $\hat{f}$. Instead of the use of inbreeding coefficients, the multiple imputation approach also can be applied to the χ^2^ statistics ($X_{i}^{2}$) of each imputed data set. Assuming the null hypothesis to be true, these can be converted into standard normal variates by

$$z_{i}=\text{sign}\left(\hat{f_{i}}\right)\sqrt{X_{i}^{2}}\;\text{with}\;\text{Var}\left(z_{i}\right)=1,$$ (6)

and these are processed again by the usual averaging and pooling rules, with *W* = 1. Because $z_{i}=\sqrt{n}{\hat{f}}_{i}$, combining χ^2^ statistics is equivalent to combining inbreeding coefficients and assuming that the variance of the inbreeding coefficient is 1/n.

#### Exact p-values:

The exact test for HWE is based on the discrete distribution of the number of heterozygotes given the allele count *n_A_* (Levene 1949; Haldane 1954; Weir 1996):

$$P\left(N_{AB}=n_{AB}|N_{A}=n_{A}\right)=\frac{n!n_{A}!n_{B}!2^{n_{AB}}}{\left(2n\right)!n_{AB}!n_{AA}!n_{BB}!},$$ (7)

The standard *p*-value for an exact test is obtained by summing the probabilities in (7) for all possible samples that are as likely or less likely under HWE than the observed sample. In this paper, we adhere to the mid *p*-value, recently proposed for use in exact HWE testing by Graffelman and Moreno (2013). The mid *p*-value is defined as *half* the probability of the observed sample plus the probabilities of all samples more extreme than the observed one (Agresti 2002). The mid *p*-value has been shown to have a rejection rate that is closer to the nominal level (Graffelman and Moreno 2013). We apply Rubin’s results for combining *p*-values from multiple imputed data sets (Licht 2010; Liublinska and Rubin 2014). Let *p_i_* be the *p*-value of the *i*th imputed data set. Then we obtain

$$z_{i}=\varphi^{-1}\left(1-p_{i}\right),$$ (8)

where *φ*^−1^ is the inverse of the distribution function of a standard normal random variable. If *p_i_* has a uniform distribution, then *z_i_* has a *N*(0,1) distribution. The *z*-statistics are averaged over the *m* imputed data sets to obtain

$${\bar{z}}_{m}=\frac{1}{m}\sum_{i=1}^{m}z_{i},\;\;W=\frac{1}{m}\sum_{i=1}^{n}\text{Var}\left(z_{i}\right)=1,$$

and from here on the usual pooling rules are applied, where we obtain the between (*B*) and within (*W*) imputation variance with equations (3) and (4), but replacing *f* by *z*. We have $\bar{z}∼t_{\nu }\left(0,T\right)$ and calculate the final multiple imputation *p*-value (*p_mi_*) as $p_{\text{mi}}=P\left(t_{v}\left(0,T\right)\geq \bar{z}\right)$. The exact *p*-value *p_i_* is often equal to one in exact tests with markers with a low MAF, giving $z_{i}=-\infty$. This would make it impossible to carry through the computations. This problem is neatly solved by using the mid *p*-value, because the latter is strictly smaller than one. If the *p*-value *p_i_* is small, *z_i_* will be large and positive. A set of small *p*-values will thus give a large ${\bar{z}}_{m}$, and *p_mi_* will tend to be small as well. Conversely a set of large *p*-values will give a large but negative ${\bar{z}}_{m}$, and this will produce a large *p_mi_*. This procedure is for one-tailed tests only. For a two-sided exact test, given only a two-sided *p*-value the correct sign of $z_{i}$ cannot be inferred. Exact tests for HWE with missings in this paper were therefore performed twice: one test for heterozygote excess and another test for heterozygote deficiency. In most practical applications, two-sided exact tests for HWE are performed, and the fact that a test that accommodates missings by combining *p*-values is one-sided is a bit of a limitation. If a two-sided exact test is required in this setting, then a pragmatic solution to this problem is to perform both one-sided tests and to calculate a two-sided multiple imputation *p*-value as *p_mi_* = 2min(*p*_mi,excess_, *p*_mi,deficiency_).

## Software

The procedures for inference on HWE with missing genotypes discussed in this paper are implemented in the R-package HardyWeinberg (https://cran.r-project.org/web/packages/HardyWeinberg/index.html). The multiple imputation part of the procedure is handled by the R-package MICE (https://cran.r-project.org/web/packages/mice/index.html).

### Data availability

The GENEVA Prematurity data are available for download from the dbGaP resource (http://www.ncbi.nlm.nih.gov/gap). They are listed as “Genome-Wide Association Studies of Prematurity and Its Complications” and the dbGaP Study Accession number is phs000103,v1.p1. Only the 1939 mothers from 3886 mother-child pairs from the Danish sample were used in this work.

## Results

In this section we describe a dataset from the GENEVA project and apply the proposed methods of inference for HWE with missing values. We first describe the dataset and then show inference on HWE using single and multiple imputation.

### Description of the dataset

**Figure 1:**
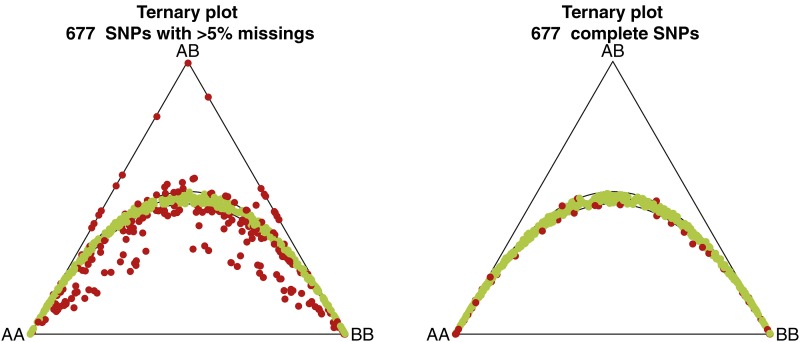
Left panel: ternary plot for 677 single-nucleotide polymorphisms (SNPs) with >5% missing. A total of 229 SNPs (34%) are significant in a χ^2^ test. Right panel: 677 SNPs without missings taken at random. A total of 56 (8%) SNPs are significant in a χ^2^ test. Significant markers are red and nonsignificant markers are green (α = 0.05).

We use a subset of SNPs from the GENEVA project on Prematurity (www.genome.gov/27550876). The original genome-wide dataset contains 657,366 SNPs typed for 3886 individuals. The dataset contains only 0.16% missing values overall, once completely missing SNPs have been eliminated. The percentage of missings per individual never exceeds 10%, and the percentage of missings per SNP never exceeds 20%, besides a subset of SNPs that was missing for all individuals. This database is filtered as follows. Only those SNPs are used that have at least 5% missing values. SNPs with a percentage of missings below this level mostly have only one or two missing values. For such SNPs, multiple imputation is unlikely to affect the statistical inference for HWE because of the large sample size used in this study. Only the mothers (female founders) in the dataset are considered, and for pairs with a first or second degree family relationship one individual was removed, in order to create a subset of independent individuals. Only autosomal SNPs are used, and the SNPs are selected to be at least 15kB apart, in order to obtain a set of approximately independent markers (Gogarten *et al.* 2012; Zheng *et al.* 2012). No thresholds for the minor allele frequencies are used. Applying these filters produces a dataset of 1939 females and 677 SNPs. Figure 1 summarizes the HWE status of the 677 SNPs and represents them in a ternary plot with the 95% acceptance region for a χ^2^ test for HWE (left panel). The figure shows that a large number of markers is out of equilibrium and reveals that disequilibrium is due mainly to a lack of heterozygotes. For comparison, we also show a ternary diagram of 677 complete SNPs that were randomly chosen from the dataset (right panel). Complete SNPs clearly show less disequilibrium.

### Imputation results

We first present a few examples of SNPs whose inference on HWE is altered by using imputation. Next, we show some results for the full subset of 677 SNPs. Three ways of dealing with missing values are considered: discarding, single imputation by SHAPEIT and multiple imputation with a multinomial logit model using flanking markers as covariates.

#### Some example SNPs:

**Figure 2:**
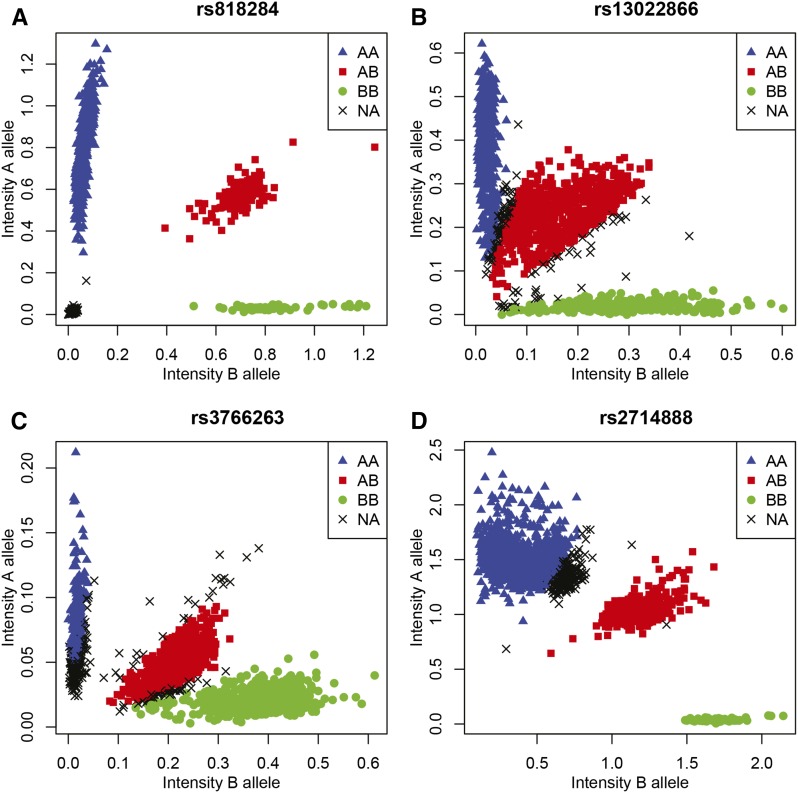
Plots of genotype calls for four single-nucleotide polymorphisms (A: rs818284; B: rs13022866; C: rs3766263; D: rs2714888) with >5% missings in the GENEVA project on prematurity.

We treat some SNPs in detail in order to show how exact inference on HWE can be affected by imputation. Figure 2 shows the plots of genotypes calls for four SNPs of the database as an example.

For these four SNPs, MI *p*-values (χ^2^ based and exact) were obtained by performing 50 imputations using a multinomial logit model with two flanking SNPs as predictors. SI *p*-values were obtained by doing a χ^2^ test and an exact test with the completed data obtained by SHAPEIT. Test results are shown in Table 1.

**Table 1:** Hardy-Weinberg equilibrium statistics for 4 SNPs with more than 5% missing values

Panel	RS	AA	AB	BB	NMV	${\hat{f}}_{\text{dis}}$	${\hat{f}}_{\text{si}}$	${\hat{f}}_{\text{mi}}$	Exact	$p_{\text{si}}\left(\chi^{2}\right)$	$p_{\text{si}}$ (ex.)	$p_{\text{mi}}\left(\chi^{2}\right)$	$p_{\text{mi}}$ (ex.)
A	rs818284	1593	138	67	141	0.451	0.458	0.451	0.000	0.000	0.000	0.000	0.000
B	rs13022866	788	781	237	133	0.046	0.012	0.015	0.046	0.596	0.571	0.525	0.526
C	rs3766263	533	865	277	264	−0.058	0.014	0.012	0.020	0.549	0.539	0.607	0.601
D	rs2714888	1092	499	69	279	0.031	0.061	0.056	0.192	0.007	0.007	0.014	0.015

RS number, genotype counts (AA,AB,BB), NMV, inbreeding coefficient under discarding ${\hat{f}}_{\text{dis}}$, inbreeding coefficients obtained by single and multiple imputation (${\hat{f}}_{\text{si}}$, ${\hat{f}}_{\text{mi}}$), two-sided exact *p*-value under discarding, two-sided exact *p*-value using SI (χ^2^ based and exact), and two-sided exact *p*-value using MI (χ^2^ based and exact). NMV, number of missing values, SNP, single-nucleotide polymorphism.

Figure 2A shows a SNP with a large number of missings close to the origin with approximately zero intensities, corresponding to null alleles. For this marker, allele intensities are of little use for imputing the missing genotypes. The inbreeding coefficients and HW *p*-values remain unaltered under SI. MI using the two closest flanking markers as covariates was hardly possible because the flanking markers had missings for almost the same set of individuals. SHAPEIT imputed almost all missings as AA genotypes, but this did not alter the HWE inference. Panel A represents a relatively common pattern for markers with missings that are out of HWE. In fact we found a successive set of more than 20 SNPs on chromosome 6 all having null alleles for the same set of individuals. Figure 2B shows a SNP with a significant deficiency of heterozygotes. Most missings border the AB cloud and are mostly imputed as heterozygotes by SI or MI. This lowers the inbreeding coefficient. An exact test for this marker is significant if the missings are discarded, but clearly nonsignificant if the missings are imputed. Figure 2C shows a SNP with a large number of missings close to the AA cluster, and also missings close to the AB cluster. An exact test that discards the missings is significant, as there is an excess of heterozygotes. Statistical imputation with information from correlated flanking SNPs mostly imputes these groups of missings as AA and AB respectively. Consequently, both exact and χ^2^-based inference is altered, the inbreeding coefficient drops toward zero when SI or MI is used, and with imputation the marker no longer deviates significantly from HWE. Figure 2D shows a SNP that would be considered in HWE when missings are discarded. A large cluster of missings bordering the AA cluster is mainly imputed as AA genotypes, and when these missings are taken into account, the inbreeding coefficient becomes larger and the marker becomes significant.

#### The full subset of SNPs:

**Figure 3:**
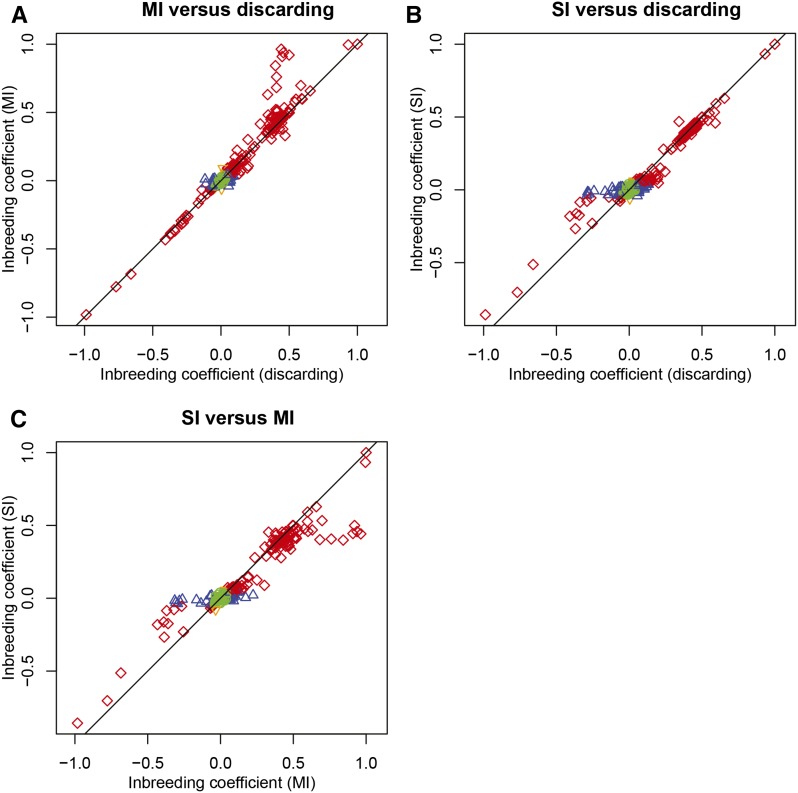
Relationships between inbreeding coefficients. (A) Estimates obtained by discarding against estimates obtained by multiple imputation using two flanking markers (MI). (B) Estimates obtained by discarding against estimates obtained by single imputation (SI). (C) SI estimates against MI estimates. Plotting symbols and colors indicate the significance of the markers in two Hardy−Weinberg equilibrium tests (α = 0.05). Red diamonds: both tests significant, green circles: both tests nonsignificant, upward blue triangles: significant in the test on the *x*-axis, nonsignificant for the test on the *y*-axis. Downward orange triangles: nonsignificant on the *x*-axis, significant on the *y*-axis.

Figure 3 shows the relationships between inbreeding coefficients obtained by discarding the missings and inbreeding coefficients obtained by SI and MI. For MI, we use a multinomial logit model that uses information from the two flanking markers: see Graffelman *et al.* (2013) for more details. For each SNP, 50 imputed datasets were created with the MICE algorithm. For some SNPs the model could not be estimated because flanking SNPs had missings for the same individuals as the SNP to be imputed, or because of perfect relationships between the SNPs involved. In these cases the allele intensities (usually having less missings than SNPs) were used as covariates instead. Imputation mostly gives similar results as discarding the missings, and estimates correlate reasonably well. However, some degree of reversal of the test results is observed. There is a set of markers (blue upward triangles) that becomes non-significant when missings are imputed (Figure 3, A and B). There is also a set of markers that is significant under MI and nonsignificant under SI (Figure 3C), showing that the results of the two imputation procedures do not always coincide. Inbreeding coefficients obtained by MI most closely resemble the inbreeding coefficients obtained under discarding. SI generally seems to decrease the inbreeding coefficients in absolute value, and so diminishes the number of markers out of HWE.

**Figure 4:**
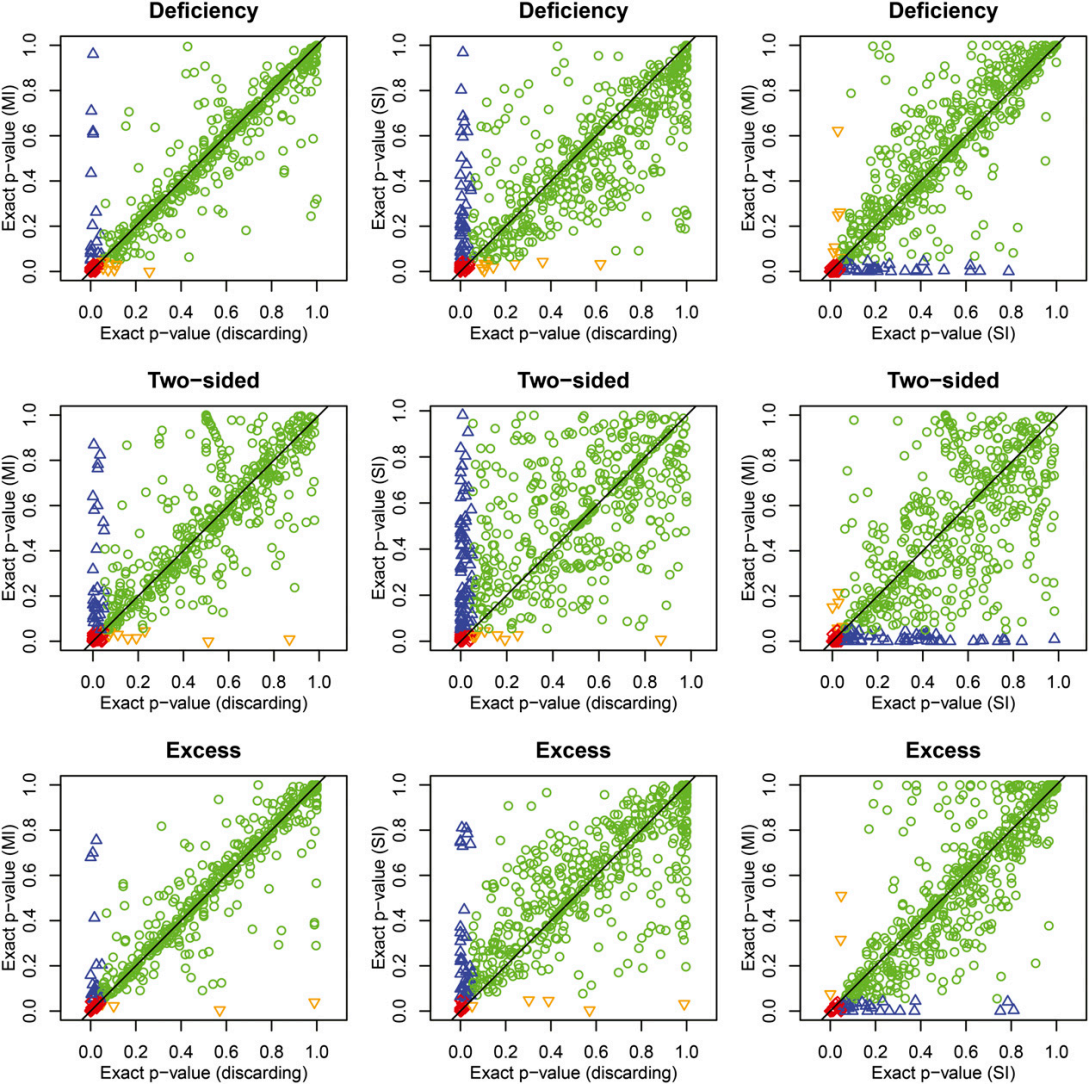
Relationships between exact *p*-values of tests for Hardy−Weinberg equilibrium (HWE) for heterozygote deficiency, excess or either (two-sided) obtained by discarding missings and imputing missings using single imputation and multiple imputation using information from two flanking markers. Plotting symbols and colors indicate the significance of the markers in two HWE tests (α = 0.05). Red diamonds: both tests significant, green circles: both tests non-significant, upward blue triangles: significant for the test on the *x*-axis, nonsignificant for the test on the *y*-axis. Downward orange triangles: non-significant on the *x*-axis, significant on the *y*-axis.

We consider the consequence of single and multiple imputation for the *p*-values of one-sided and two-sided exact tests for HWE. The relationships between *p*-values obtained by discarding and imputation of missings are plotted in Figure 4.

The plots of the *p*-values give some idea of the performance of the SI and MI approach. In general, *p*-values obtained by MI resemble the *p*-values obtained by discarding missings, in particular for the nonsignificant markers (first column of Figure 4). The *p*-values obtained by SI generally show larger differences with respect to discarding missings (second column of Figure 4). A considerable subset of markers becomes nonsignificant when missings are imputed by SI or MI, as shown by the vertical blue stripe in the first two columns of Figure 4. A few markers become significant upon imputation. If we take the two-sided test as a reference, then 33 SNPs (4.9%) turned nonsignificant under MI, whereas nine (1.3%) turned significant under MI. SI using SHAPEIT produced larger changes: 79 SNPs (11.7%) turned nonsignificant under SI, whereas 7 (1%) turned significant under SI. In total about 6% of the test results changed under MI, and about 13% under SI.

**Figure 5:**
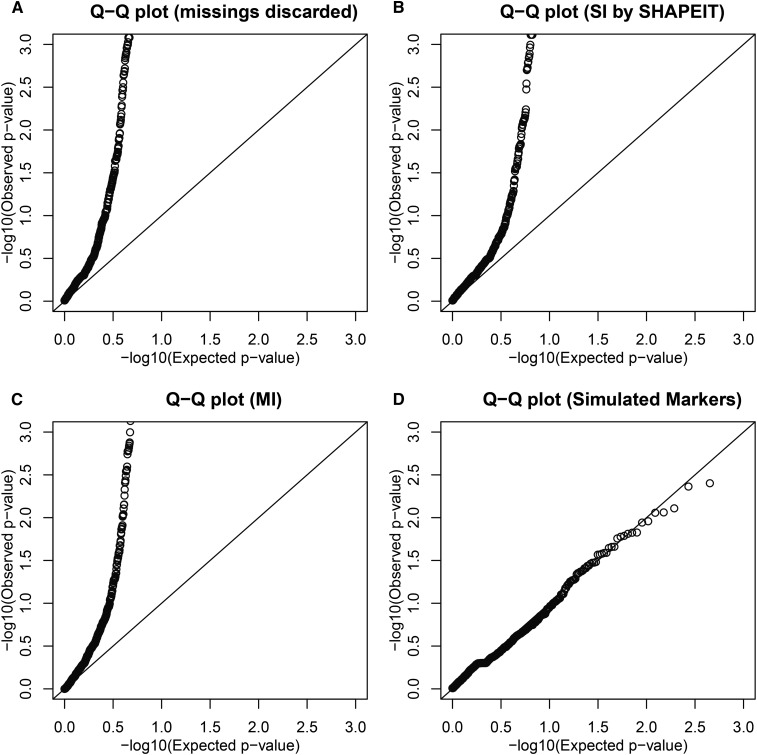
Q-Q plots of *p*-values for tests for Hardy−Weinberg equilibrium obtained by (A) discarding missings, (B) single imputation of missing values and (C) multiple imputation. (D) shows a reference Q-Q plot of the *p*-values for a dataset of 677 simulated SNPs with the same sample size and allele frequency distribution as the observed data.

The Q-Q plots of the *p*-values using a uniform reference distribution are shown in Figure 5. A logarithmic scale is used to emphasize the lower tail of the distribution. Figure 5D shows the expected pattern for the database under HWE and can be used as a reference graph. These graphs confirm that there are many more significant results than would be expected by chance alone. SI is seen to slightly improve the distribution of the *p*-values. None of the methods used yields a uniform *p*-value distribution, most likely because the studied subset of SNPs is not only subject to missing observations but also to considerable genotyping error.

## Discussion

This paper presents an exact test for HWE that takes missing genotypes into account. For small samples or low minor allele frequencies, the exact test for HWE generally is preferred over the classical χ^2^ test. Thus, the work presented here allows one to test HWE in small samples or low MAF samples by an exact procedure even if there is missing genotype information. The mid *p*-value is used as the test-statistic to be used in the exact test for HWE. We note that the mid *p*-value has the property that its value in a test for heterozygote excess is one minus the *p*-value obtained in a test for heterozygote deficiency. The standard one-sided exact *p*-value does not have this property. It this sense testing is akin to a student *t*-test for quantitative variables, where the *p*-value for the one-sided test with *H*_0_: μ = μ_0_ against *H*_1_: μ > μ_0_ is also one minus the *p*-value for the one-sided test with $H_{1}:\text{μ}\leq {\text{μ}}_{0}$. This property directly carries over to the *p*-value obtained by multiple imputation (*p_mi_*). Thus, if one wants to do both one-sided tests, only the exact test for a single one-sided test needs to be calculated. This means that doing both tests does not increase the computational burden in comparison with a standard two-sided test. We also note that the proposed two-sided multiple imputation *p*-value (*p_mi_*) cannot exceed one, precisely because *p*_mi,excess_ = 1 − *p*_mi,deficiency_. At least for large samples, the two-sided *p_mi_* is seen to correlate well with the two-sided *p*-values obtained by combining inbreeding coefficients. In relation with this, we note that Q-Q plots of standard exact *p*-values made against the uniform distribution as a reference distribution are often used and typically show a band of *p*-values at the value 1 (Rohlfs and Weir 2008). A *p*-value of 1 occurs if the observed sample is the most likely one for the given minor allele count. This often happens for markers with a low MAF. If the mid *p*-value is used, this band of *p*-values at 1 disappears, because the probability of the most likely sample is halved and a wider range of *p*-values can occur.

Markers that strongly deviate from HWE have, especially for large samples like the one studied here, *p*-values that are vanishingly small, leading to $z_{i}=\infty$[see Equation (8)]. To avoid computational problems such *p*-values were set to the smallest floating point number that makes $p_{i}$ different from 0. Likewise, *p*-values whose difference from 1 is vanishingly small were set to 1 minus the smallest floating point number that makes (1 − *p_i_*) different from 1. This guarantees that the multiple imputation algorithm produces a *p*-value close to 0 if all imputed data sets are highly unlikely under HWE, and a *p*-value close to 1 if all imputed data sets are highly likely under HWE.

### Implications for genome-wide association studies

Data cleaning for genome-wide association studies routinely uses HWE testing to filter out SNPs of low quality (Laurie *et al.*, 2010). Because HWE generally is expected in human populations, departures from HWE are expected to indicate problems with the data rather than some biological phenomenon. However, if SNPs that deviate significantly from HWE are eliminated routinely before association analysis, there may be a loss of power for association tests. It is well known (*e.g.*, Nielsen *et al.* 1998) that the HWE test at genetic markers, when confined to cases or to controls, is actually a test for linkage disequilibrium between the marker and the disease genes. Eliminating markers that fail HWE tests also may be eliminating SNPs associated with the disease. We suggest that a goal should be to retain as many markers as possible in the association analysis and maybe focus HWE tests on those markers that do appear to be associated with the disease. Markers found to be significantly associated with a trait could be examined for the questions: 1) Do they have low MAF? 2) Are they out of HWE? 3) Do they have a large number of missings? 4) Do the genotype call plots look unusual? These questions are, in fact, part of sound analysis pipelines.

On the other hand, many markers of the GENEVA subset studied in this paper (more than 5% have missing data) have “bad” genotype calling plots and 34% of the subset markers are out of HWE. Most of these are likely out of HWE due to genotyping error. If a marker presents disequilibrium AND it has a high missing rate, genotyping error is probably the most likely explanation for both things. But if a marker presents disequilibrium without a high missing rate then there is less evidence for genotyping error and it may be appropriate to keep the marker for association analysis.

### GENEVA data results

The analysis of the GENEVA database on prematurity shows that disequilibrium is more often due to a deficiency of heterozygotes than to an excess of heterozygotes. In 79% of the cases of statistically significant disequilibrium, the latter was due to a deficiency and in 21% of the cases it was due to an excess of heterozygotes. That disequilibrium is more often due to heterozygote deficiency seems to be a characteristic of SNP data (Graffelman *et al.* 2013). An explanation for this is that heterozygous genotypes have a greater probability of being missing or being misclassified as a homozygote, basically because in the intensity plots they form a middle cloud bordering two other groups. Homozygote genotypes maybe misclassified as heterozygotes but it is less likely that a homozygote AA is misclassified as a BB or the reverse. If the sample is large, and all three genotypes are present with substantial frequencies, then an AB genotype may be expected to have double the misclassification rate and the missingness rate of an AA genotype. A few monomorphic markers with a considerable number of missings were observed. Such markers could potentially be tested for HWE if missings are taken into account and imputed with genotypes that possibly differ from the single observed homozygote. MI with the multinomial logit model was not possible in these cases because the response shows no variation. SHAPEIT assigned the single observed common homozygote to all missings in these cases. However, in the call plot sometimes the missings clustered outside the cloud of the common homozygote, indicating that a different genotype might indeed exist. This stresses the need for an imputation method that can impute genotypes that have not been observed in the sample.

A considerable set of markers had a zero count for one homozygote. In some cases missings formed a separate cluster in the call plot, separate from the two observed genotypes, that most likely corresponded to the unobserved homozygote. This generates a situation of (false) heterozygote excess. In this situation, the MI approach with the multinomial logit model is not able to create sensible imputations, because it can impute only the two observed categories in the data. In some instances, SHAPEIT did impute the missings as the second homozygote and could diminish heterozygote excess. The fact that SHAPEIT can impute missings with genotypes that have not been observed in the data is an advantage and it explains at least in part that the inbreeding coefficients obtained with this method tend to be closer to zero than those obtained by MI. Single-imputation programs sometimes produce a best-guess value for a missing value using the genotype that has the highest posterior probability. Using the full vector of posterior probabilities for all genotypes could improve the inference for HWE, as it would take better account of imputation uncertainty. This requires a test for HWE that is based on probabilities instead of on genotype counts. The development of such a test is part of our ongoing research.

The strength of the MI approach described in this paper lies in two points: First, MI takes uncertainty in the imputations into account by imputing many times. Second, only a few correlated variables (flanking markers and/or intensities) are needed for imputation. This is an advantage in situations where no reference panels are available, or when there are not sufficient markers for reliable haplotype estimation. A future modification of the multinomial logit model that allows for the imputation of all three genotypes would form a valuable extension of the work presented here.

We comment on some additional aspects of the call plots of studied subset of the GENEVA database. Many call plots of the significant markers were of poor quality due to null alleles, cluster overlap, or more than three clusters. Missing genotypes often correspond to null alleles (intensities close to zero). Markers that became significant upon imputation often showed additional clusters of missings separated from the AA, AB, and BB clouds. Both imputation algorithms used here are restrictive in the sense that they force such missings to be imputed as one of the three habitual genotypes and do not account for the existence of null alleles or copy number variation.

The database on prematurity studied in this paper has a low overall percentage of missing values and concerns a large sample. Most SNPs have only a few missing values, and inference for HWE is hardly affected by discarding or imputing the missings. Accounting for missingness becomes more interesting if the number of missings is substantial because then there is more scope for bias if missings are ignored. If no MAF threshold is applied, then the set of markers studied here contains precisely those markers generally considered poor markers. For such markers HWE is rejected more often, in particular if the missings are discarded. Often this subset of markers is ruled out from analysis by applying thresholds for the MAF and the HWE exact *p*-value (frequently used exclusion criteria are MAF below 0.05 and HWE exact *p*-value below 0.001). It is precisely for this subset that single and multiple imputation can provide improved statistical inference.
